# Investigation of voriconazole heteroresistance in clinical isolates of *Trichosporon asahii* from a multicenter study in China

**DOI:** 10.1128/spectrum.00109-25

**Published:** 2025-06-20

**Authors:** Chenlu Liu, Qiaoying Gao, Yingxing Li, Jinhan Yu, Shuying Yu, Xinfei Chen, Xue Li, Yingchun Xu, Ying Zhao, Lina Guo

**Affiliations:** 1 Department of Laboratory Medicine, Peking Union Medical College Hospital, Chinese Academy of Medical Sciences and Peking Union Medical College 670119, Beijing, China; 2 State Key Laboratory of Complex, Severe, and Rare Diseases, Chinese Academy of Medical Sciences and Peking Union Medical College, Beijing, China; 3 Graduate School, Peking Union Medical College, Chinese Academy of Medical Sciences 12501, Beijing, China; 4 Clinical Laboratory, Nankai Hospital Affiliated to Tianjin Medical University, Beijing, China; 5 Biomedical Engineering Facility of National Infrastructures for Translational Medicine, Institute of Clinical Medicine, Peking Union Medical College Hospital, Peking Union Medical College, Chinese Academy of Medical Sciences 12501, Beijing, China; Institut Pasteur, Paris, France

**Keywords:** *Trichosporon asahii*, heteroresistance, voriconazole, susceptibility, cross-resistance

## Abstract

Heteroresistance refers to the presence of subpopulations within seemingly homogeneous microbial cells that exhibit varying sensitivities to antifungal agents, potentially contributing to treatment failure. Our study investigated the heteroresistance levels of clinical isolates of *Trichosporon asahii* collected from a multicenter study in China. A total of 62 isolates from 31 research centers, representing five different genotypes, were analyzed. We assessed the minimal inhibitory concentrations (MICs) of voriconazole (VRC), the level of heteroresistance to voriconazole (LHV), the strains’ capacity for adaptation to high VRC concentrations (ADP), and the stability of the heteroresistance phenomenon. The isolates had low VRC MICs (average 0.038 µg/mL), with 95.2% (59/62) classified as wild type. Heteroresistance to VRC was observed in all isolates, with an average LHV (0.25–4 µg/mL) 23 times higher than the MICs (0.012–0.19 µg/mL). Additionally, 90.3% of strains tolerated VRC concentrations at least 16 times the MICs, with heteroresistant subpopulations appearing at frequencies ranging from 0.002% to 0.830%. Furthermore, the strains rapidly adapted to elevated drug concentrations following brief exposure to VRC, with 71.0% tolerating VRC at 8 µg/mL and an average ADP value 285 times the MICs. However, the strains gradually lost this resistance when serially passaged on drug-free SDA plates, with the time required for resistance loss varying among strains. Our study is the first to reveal the presence of heteroresistance in clinical *Trichosporon asahii*, adding to the body of research on fungal heteroresistance and posing challenges to the clinical interpretation of MIC values for guiding treatment.

**IMPORTANCE:**

*Trichosporon asahii* has become an increasingly important fungal pathogen responsible for invasive infections in clinical settings. Due to its natural resistance to flucytosine and echinocandin classes, azole drugs, especially voriconazole, are the mainstay of treatment. The phenomenon of heteroresistance may be associated with treatment failure in fungal infections; however, this phenomenon remains unclear in *T. asahii*. Our study provides the first comprehensive characterization of voriconazole heteroresistance in *T. asahii,* significantly advancing our understanding of antifungal resistance dynamics in this emerging pathogen. Our findings also challenge the reliability of conventional minimal inhibitory concentration-based therapeutic guidance for *T. asahii* infections.

## INTRODUCTION

*Trichosporon* is a yeast-like basidiomycete and a clinically rare opportunistic pathogenic fungus (1), which is widely distributed in the environment and can also be part of the normal skin flora in humans. Occasionally, it is found as part of the normal gastrointestinal or upper respiratory tract microbiota. *Trichosporon* colonies are white or cream-colored with a gyrate appearance, and blastoconidia, arthroconidia, pseudohyphae, and true hyphae can be observed via microscope (2–4). The genus *Trichosporon* is considered an important cause of white piedra, summer-type hypersensitivity pneumonitis, and several kinds of life-threatening fungal invasive infections (5). Invasive infections caused by *Trichosporon* are commonly observed in immunocompromised and immunodeficient patients, as well as those with hematological disorders (1, 6, 7). In 2015, the genus *Trichosporon* underwent significant taxonomic revisions (8). *Trichosporon asahii*, the causative agent of Trichosporonosis, and *Trichosporon inkin* and *Trichosporon ovoides*, the causative agents of white piedra, were retained, while some species were reassigned to other genera (e.g., *Cutaneotrichosporon dermatis* and *Apiotrichum mycotoxinivorans*).

In recent years, the incidence of *T. asahii* infections has increased significantly, making it the leading fungal genus in invasive yeast infections after *Candida* and *Cryptococcus* in Asia, with a reported mortality rate as high as 80% in immunocompromised patients (1, 9). In terms of pharmacological treatment, due to the inherent resistance to echinocandins, azoles have become the primary therapeutic class for invasive *Trichosporon* infections. Voriconazole is a first-line therapy demonstrating strong *in vitro* activity against most *Trichosporon*, with excellent *in vivo* efficacy observed in the majority of clinical and animal studies (9, 10).

Heteroresistance was first described in the 1940s (11), and its most widely accepted definition refers to a heterogeneous bacterial population containing one or several subpopulations that exhibit higher levels of antibiotic resistance compared to the main population (12). These resistant subpopulations can adapt to increasing drug concentrations progressively (13, 14). Heteroresistance may represent a mechanism by which bacteria and fungi adapt to environmental pressures (15). Due to their instability, low frequency, and transient nature, the detection and study of these subpopulations pose significant challenges, often complicating the clear classification of bacteria and fungi as either susceptible or resistant. *In vitro* experiments, mathematical models, animal infection models, and clinical studies have shown that during antibiotic exposure, resistant subpopulations can rapidly proliferate and become dominant due to selective pressure, finally leading to treatment failure (16, 17). In recent years, heteroresistance has gained increasing attention and research interest, and it has also been identified in fungi such as *Candida glabrata* (18, 19) and *Cryptococcus neoformans* (20, 21).

Up to now, there is no standardized method for detecting heteroresistance, and the methodologies employed vary across different laboratories. The most commonly used methods include the E-test, disk diffusion assay, and population analysis profiling (PAP). Among these, PAP is considered the most reliable method for identifying heteroresistance, but it is costly and labor-intensive, making it unsuitable for routine clinical use. In contrast, other methods, such as E-test and disk diffusion assay, exhibit lower sensitivity and specificity, with high rates of false negatives and false positives, leading to reduced reliability of results (16, 22). Currently, there is a need for the development of new diagnostic methods in the clinical setting to detect low-frequency resistant cells, while ensuring high sensitivity, reproducibility, and low complexity (23). In this study, the PAP method was employed for the detection of heteroresistance.

Asia, particularly China, is the main source of clinical cases involving *T. asahii* (1). However, there have been no studies on heteroresistance in *T. asahii*. This study is the first to globally reveal the heteroresistance against voriconazole as recorded in clinical *T. asahii* isolates, providing valuable insights into likely reasons for therapeutic failure in *T. asahii* infection patients.

## MATERIALS AND METHODS

### Study design and definitions

*Trichosporon* isolates were collected between 2009 and 2021 from patients enrolled in the CHIF-NET study (24), which was a prospective, laboratory-based, multicenter study of invasive yeast infections. Invasive fungal infections were defined according to the consensus statement of the Invasive Fungal Infections Cooperative Group of the European Organization for Research and Treatment of Cancer and the Mycoses Study Group of the National Institute of Allergy and Infectious Diseases (25).

### Isolates

The study included a total of 62 clinical *T. asahii* isolates, collected from 31 centers across 13 provinces, 1 autonomous region, and 4 municipalities in the following administrative divisions of China: 24 isolates from North, 13 from Central South, 9 from East, 6 from Northwest, 6 from Northeast, and 5 from Southwest. The majority of isolates were derived from blood (24/62, 38.71%), ascitic fluid (11/62, 17.74%), and catheters (8/62, 12.90%). A smaller proportion was obtained from abscesses (4/62, 6.45%), sputum (3/62, 4.84%), bronchoalveolar lavage fluid (3/62, 4.84%), cerebrospinal fluid (3/62, 4.84%), and other specimens.

### Molecular identification and IGS1 genotyping

The isolates were identified to the species level by sequence analysis of the IGS1 region using primer pairs (26). The sequencing results of the IGS1 region of the experimental strains were aligned with known genotypes of *T. asahii* from the GenBank database using the MEGA11 software for multiple sequence alignment. Genotyping of the experimental strains was performed using the UPGMA method.

### Antifungal susceptibility testing

The minimum inhibitory concentration (MIC) for voriconazole (VRC) was determined by the E-test method after 48 hours of incubation (27, 28). *Candida krusei* ATCC 6258 and *Candida parapsilosis* ATCC 22019 were used as quality control strains in the experiment. Since species-specific clinical breakpoints for *T. asahii* have not been established, epidemiological cutoff values were applied for interpreting susceptibility testing results. Isolates demonstrating MIC values ≤ 0.125 µg/mL were classified as wild-type strains (17).

### Level of heteroresistance to voriconazole

**Fig 1:**
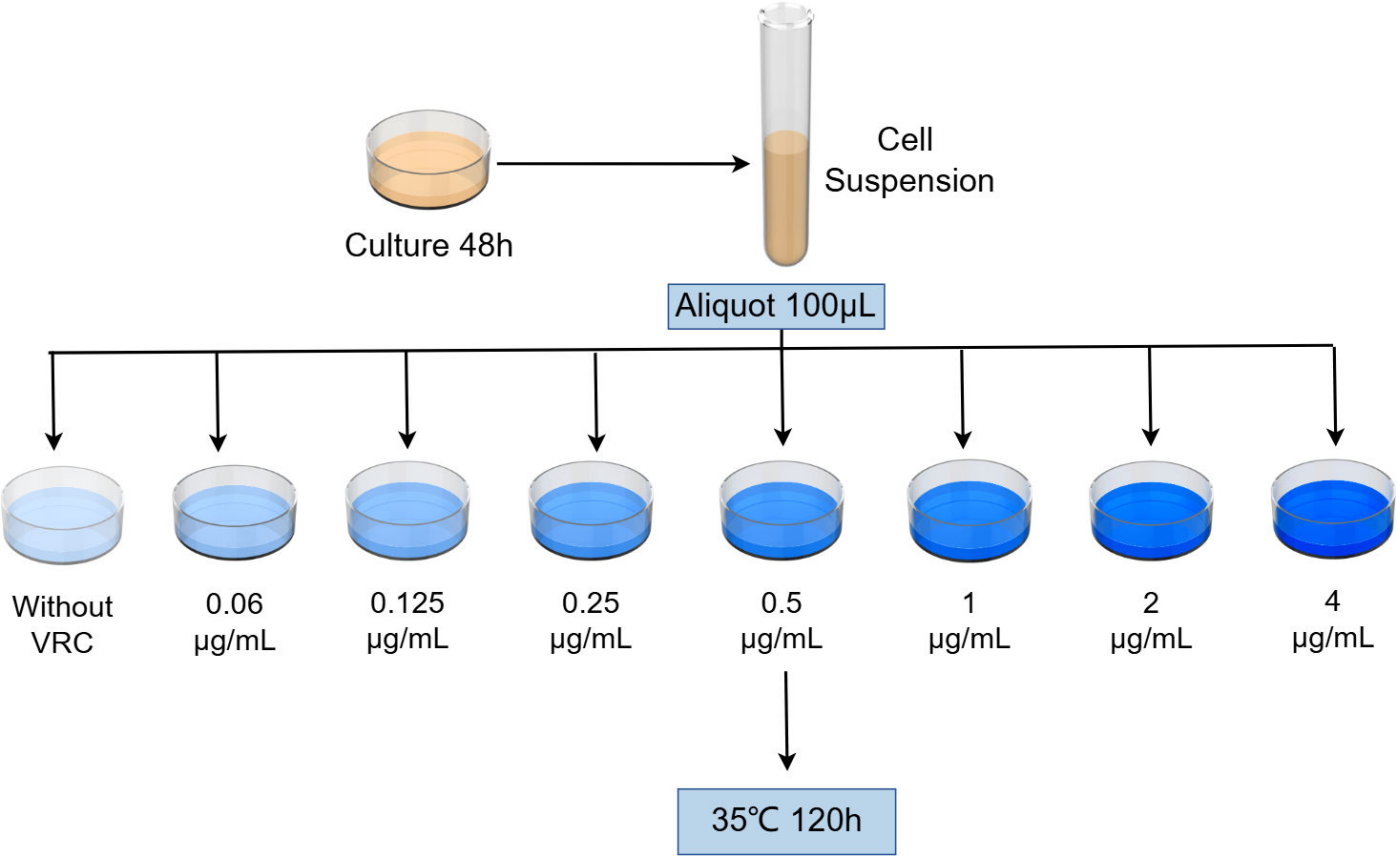
Procedure for testing the level of heteroresistance to voriconazole.

Level of heteroresistance to voriconazole (LHV) was determined using population analysis profiling on SDA plates containing different concentrations of voriconazole (0.06–4 μg/mL) (Fig. 1). The criterion for defining heteroresistance in our study was the presence of a small resistant subpopulation within the strain, where these fungal cells are capable of growing at drug concentrations at least eight times higher than the MIC for the majority of sensitive cells in the population, with the frequency of resistant subpopulations ranging between 10^−2^ and 10^−7^ (29). The lowest concentration at which such resistant subpopulations appear was determined as each strain’s LHV.

The number of cells in each culture was determined by measuring the McFarland turbidity of culture dilutions in sterile saline using a bioMérieux turbidimeter (DensiCHEK Plus). Cell suspensions were adjusted to a density of 1 × 10^6^ CFU/mL in sterile saline, and 100 µL was added to the drug-containing plates, followed by spreading the suspension evenly until fully absorbed. After incubating at 35°C for 120 hours, colony growth was documented photographically, and the percentage of resistant subpopulations was calculated.

### Adaptive LHV (ADP)

To determine the maximum drug concentration tolerated by the strains, the VRC concentration on the plates was gradually increased, beginning with the LHV of the strain as the initial plate concentration. The heteroresistant subpopulation grown on the plate was then mixed thoroughly. Subsequently, 100 µL suspension of each isolate (1 × 10^6^ CFU/mL) was plated on agar plates containing increasing concentrations of VRC, and the plates were incubated at 35°C for 120 hours before recording the results. SDA agar plates without voriconazole were used as positive control media for fungal growth.

**Fig 2:**
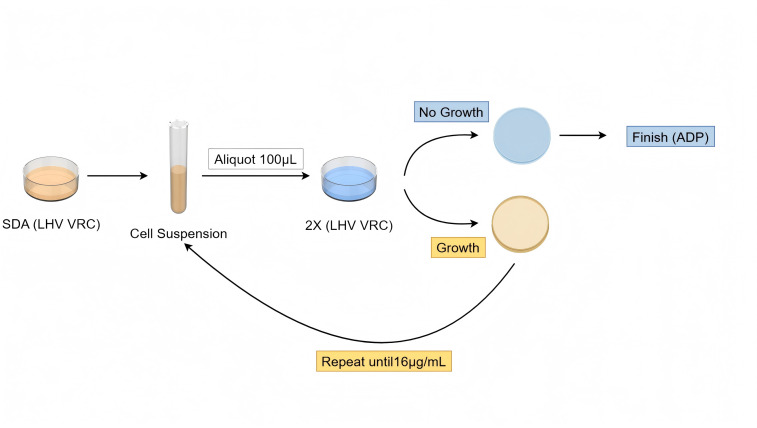
Procedure for the adaptation test at levels higher than the strain’s LHV.

If the colonies could tolerate the current concentration, the VRC concentration on the plate was doubled. This process was repeated until the VRC concentration reached 16 µg/mL or no growth was observed. The highest concentration at which the strain could continue to grow was recorded as the strain’s adaptation concentration (ADP). For isolates that did not adapt to concentrations higher than the initially recorded level of heteroresistance against voriconazole (LHV), this LHV value itself was considered as the highest concentration of VRC at which the isolate was able to grow (Fig. 2).

### Stability of ADP

The stability of resistance to high concentrations of voriconazole was evaluated. Our study selected three voriconazole-resistant colonies for resistance stability testing: Y169ADP8-L3, Y169ADP16-L4, and Y158ADP16-L3. The strains were passaged onto drug-free SDA plates every 2 days, and the MIC values were determined at each passage using four E-test strips (VRC, posaconazole [POS], fluconazole [FLU], and itraconazole [ITC]) to assess changes in sensitivity. The approach aimed to investigate whether the isolates would revert to their initial sensitivity in the absence of drug pressure.

### Statistical analysis

Statistical analysis and graphing were carried out using GraphPad Prism 9.5.1, with significance accepted at *P* < 0.05. After normality and homogeneity of variance tests, either parametric or non-parametric tests were selected based on the data type. Group comparisons were conducted using Analysis of Variance and/or the Kruskal-Wallis test. For Kruskal-Wallis testing, Dunn’s *post hoc*test with *P* value adjustment was applied. Experimental flowcharts were created using Figdraw software (ID: ORYYYaa6aa, RSITU1e9e2).

## RESULTS

### Strain identification and genotype distribution

The 62 clinical isolates included in our study were confirmed as *T. asahii* through MALDI-TOF and IGS1 region sequencing analysis. Among the clinical isolates, five genotypes were identified (Table 1), with genotypes 1 (22/62, 35.5%), 4 (21/62, 33.9%), and 3 (17/62, 27.4%) being the most prevalent. Genotypes 5 and 7 were each represented by a single isolate, found in Hubei Province (Central South region) and Guizhou Province (Southwest region), respectively.

**TABLE 1:** Distribution of IGS1 genotypes and MIC of VRC among 62 T. *asahii* isolates

Characteristic	Total no.	No. of isolates with indicated genotype				
		Genotype 1 (*n* = 22)	Genotype 3 (*n* = 17)	Genotype 4 (*n* = 21)	Genotype 5 (*n* = 1)	Genotype 7 (*n* = 1)
Region of China						
North	23	4	6	13		
Central	13	4	3	5	1	
Eastern	9	4	2	3		
Northwest	6	6				
Northeast	6	3	3			
Southwest	5	1	3			1
MIC						
0.012	2		1	1		
0.016	10	3	2	4	1	
0.024	23	7	3	12		1
0.032	17	7	6	4		
0.048	3	2	1			
0.064	2	2				
0.094	1		1			
0.125	1		1			
0.19	3	1	2			

### Level of heteroresistance to voriconazole

The experimental results of the 62 clinical strains are presented in Table 2. Heteroresistance was observed in all clinical strains, suggesting that heteroresistance may be an inherent characteristic of the strains. The level of voriconazole heteroresistance in *T. asahii* ranged from 0.25 to 4 µg/mL. The majority of strains (90.3%) were able to grow on VRC plates at concentrations 16 times or more the MIC value, with one strain (Y63) even capable of tolerating voriconazole at concentrations 63 times the MIC. The percentage of cells capable of growing at increased voriconazole concentrations, referred to as the “frequency of heteroresistance in %,” was less than 1% for all resistant subpopulations in this study, with frequencies ranging from 0.002% to 0.830%.

**TABLE 2:** MIC values, LHVs, LHV/MIC ratio, proportion of heteroresistance (%), and ADP in clinical isolates of *Trichosporon asahii*

Strain	MIC (μg/mL)	LHV (μg/mL)	Multiply	Proportion of heteroresistance (%)	ADP (μg/mL)
1	0.032	0.5	16	0.08	8
3	0.048	1	21	0.076	2
4	0.016	0.25	16	0.29	1
5	0.024	0.25	10	0.83	16
6	0.024	0.5	21	0.12	8
10	0.016	0.25	16	0.1	4
11	0.024	0.25	10	0.372	16
12	0.032	1	31	0.008	8
13	0.032	0.5	16	0.332	8
14	0.032	0.5	16	0.324	16
15	0.024	0.5	21	0.024	16
18	0.016	0.5	31	0.004	1
20	0.032	1	31	0.06	16
21	0.024	0.5	21	0.004	8
22	0.032	1	31	0.604	2
24	0.024	0.5	21	0.012	8
26	0.024	0.5	21	0.004	4
28	0.024	0.5	21	0.19	8
29	0.024	0.5	21	0.014	16
30	0.032	0.5	16	0.344	16
31	0.012	0.5	42	0.012	16
32	0.024	0.5	21	0.012	16
35	0.19	2	11	0.016	16
38	0.024	0.5	21	0.038	16
42	0.024	0.25	10	0.138	8
43	0.024	0.5	21	0.01	16
45	0.032	0.5	16	0.234	16
46	0.024	1	42	0.194	16
48	0.016	0.5	31	0.05	16
51	0.024	0.5	21	0.328	2
55	0.016	0.5	31	0.012	16
58	0.024	0.5	21	0.01	8
61	0.016	0.5	31	0.006	16
62	0.016	0.5	31	0.23	2
63	0.016	1	63	0.012	16
65	0.024	0.5	21	0.014	16
66	0.024	0.5	21	0.002	16
67	0.016	0.5	31	0.01	16
69	0.032	0.5	16	0.004	16
70	0.064	1	16	0.012	16
72	0.024	0.5	21	0.002	16
73	0.016	0.5	31	0.012	8
77	0.024	0.5	21	0.038	1
79	0.024	0.5	21	0.014	1
82	0.032	0.5	16	0.078	16
101	0.064	2	31	0.036	4
103	0.094	2	21	0.034	16
110	0.032	1	31	0.08	16
114	0.19	2	11	0.05	4
122	0.048	1	21	0.07	4
124	0.032	1	31	0.02	4
158	0.125	4	32	0.05	16
160	0.048	0.5	10	0.222	16
168	0.024	0.5	21	0.344	8
169	0.19	4	21	0.06	16
172	0.032	0.5	16	0.014	4
218	0.032	0.5	16	0.128	16
220	0.024	0.5	21	0.076	4
223	0.032	0.5	16	0.098	4
224	0.032	0.5	16	0.03	16
227	0.012	0.5	42	0.014	4
230	0.032	1	31	0.048	8

The LHV of strains with the same MIC values was analyzed, and the results are shown in Table 3. We observed that there are differences in the level of heteroresistance to voriconazole among strains with identical MIC values. Additionally, higher MIC values were associated with higher LHV, although this relationship is not strictly linear. Moreover, we observed that when exposed to the same concentration of VRC, partial strains with lower MIC values exhibited similar tolerance to antifungal pressure as strains with higher MIC values. For instance, strains with MIC values ranging from 0.016 to 0.064 μg/mL displayed the same 1 µg/mL LHV, suggesting that LHV may play a significant role in assessing a strain’s resistance to external environmental pressures.

**TABLE 3:** Integrated data of strain MIC values and LHVs

MIC	LHV	No. of isolates (%)	Heteroresistance (%)
0.012	0.5	2 (100)	100
0.016	0.25	2 (20)	
	0.5	7 (70)	
	1	1 (10)	
0.024	0.25	3 (13.0)	
	0.5	19 (82.6)	
	1	1 (4.3)	
0.032	0.5	11 (64.7)	
	1	(35.3)	
0.048	0.5	1 (33.3)	
	1	2 (66.7)	
0.064	1	1 (50)	
	2	1 (50)	
0.094	2	1 (100)	
0.125	4	1 (100)	
0.19	2	2 (66.7)	
	4	1 (33.3)	

### Adaptive LHV

To address the isolates’ ability to adapt to higher voriconazole concentrations than those recorded as LHV, we increased the VRC concentration in the plate gradually. Since the strains were only passed once on VRC plates before observing results, the adaptive test reflects the strains’ short-term adaptation ability to high drug concentrations. It was found that 71.0% (44/62) of the strains could rapidly acquire the ability to tolerate voriconazole at a concentration of 8 µg/mL, and 51.6% (32/62) of the strains could grow on plates containing ultra-high concentrations (16 µg/mL) of voriconazole.

**Fig 3:**
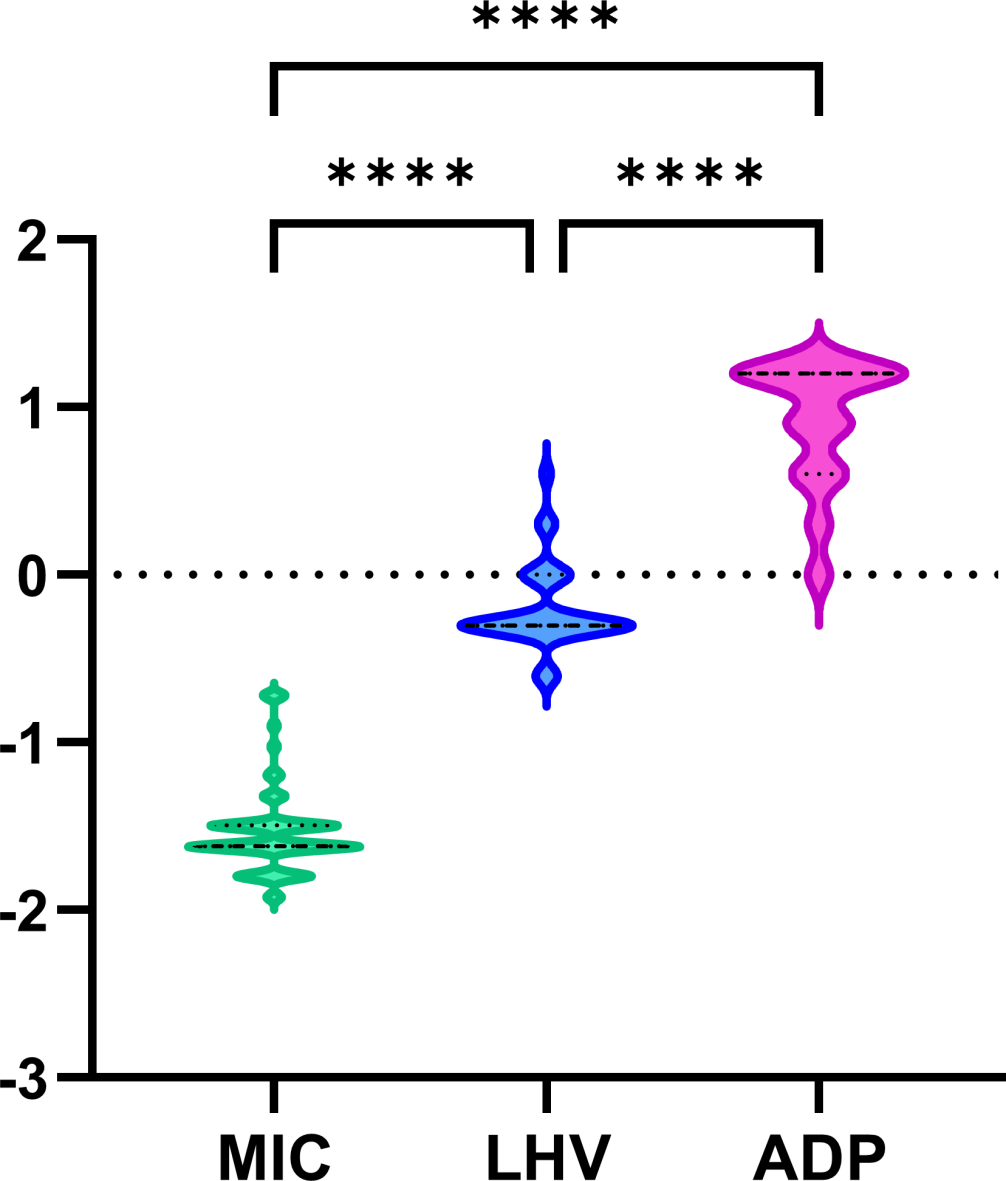
Violin plot: comparison of the values of MIC, LHV, and ADP in µg/mL in clinical strains. The comparison was performed by applying non-parametric Kruskal-Wallis testing after transformation to natural logarithm values and with significance accepted at *P* < 0.05. LN, natural logarithm; – –, median values; ……, 25th or 75th percentile; and ****, *P* < 0.0001.

The mean values of MIC, LHV, and ADP assays were compared using statistical testing with data transformed to natural logarithm values (Fig. 3). Significant differences were observed among the MIC, ADP, and LHV values of the clinical isolates (*P* < 0.0001). The LHV was on average 23 times the MIC, and the ADP was on average 285 times the MIC, indicating that the strains have a strong adaptive regulatory capability against antifungal agents.

### Cross-resistance to other antifungal agents

Previous studies have demonstrated that *Cryptococcus neoformans* strains, which develop adaptive resistance to fluconazole, can also exhibit cross-resistance to amphotericin B and flucytosine, even without prior exposure to these drugs (30). The ability to develop cross-resistance without direct contact has garnered significant attention. Our study similarly evaluated the MIC values for voriconazole, posaconazole, fluconazole, and itraconazole in the original isolates and single colonies randomly selected from plates containing different concentrations of VRC.

Table 4 presents the MIC values for selected strains and their heteroresistant colonies isolated from VRC plates. The results indicated that compared to the original strains, the majority of colonies exhibited significantly increased MIC values for all four antifungal drugs, while a small portion of colonies showed either unchanged or slightly decreased MIC values. Furthermore, variations in MIC values were observed among colonies isolated from the same VRC plate, with some colonies capable of tolerating extremely high VRC concentrations. These findings further corroborate the existence of heteroresistance.

**TABLE 4:** Comparison of antifungal susceptibilities of parent isolates and their heteroresistant clones to four agents (VRC, FCA, POS, and ITR) in µg/mL^*a*^

Strain	Isolate or clone	Antifungal drug MIC (μg/mL)			
		VRC	FCA	POS	ITR
Y158	Parent	0.125	128	2	1
	LHV4-L1	0.25	>256	2	2
	LHV4-L4	0.19	>256	3	3
	ADP8-L2	0.38	>256	4	16
	ADP8-L4	0.75	>256	6	16
	ADP16-L1	1	>256	>32	>32
	ADP16-L2	1	>256	>32	>32
Y169	Parent	0.19	>256	1	1
	ADP8-L1	8	>256	32	>32
	AD16-L2	32	>256	32	>32
Y172	Parent	0.032	3	0.38	0.5
	LHV0.5-L3	0.032	4	0.5	0.38
	ADP2-L4	0.094	48	4	6
Y15	Parent	0.024	2	0.5	0.25
	ADP0.5-L2	1	>256	32	>32
	ADP0.5-S3	0.064	24	2	0.75
Y160	Parent	0.048	6	0.5	0.5
	LHV1-L2	0.5	48	4	2

^*a*^ The strain naming convention follows the pattern of strain number + LHV/ADP + L/S number, where L, large colony; S, small colony; LHV, colony derived from the first drug-containing plate; and ADP, strain derived from the adaptive experiment drug-containing plate. For example, Y169ADP8-L3 refers to the large colony, number 3, that adapted to a voriconazole concentration of 8 μg/mL.

We also observed that as the strain’s tolerance to VRC increases, its resistance to the other antifungal agents also increases gradually. Moreover, the fold increase in MIC values for the resistant subgroups of POS and ITR is even more significant than that of VRC in Y158. Surprisingly, the strain was able to acquire resistance to ITR and POS without prior exposure to them, which warrants clinical attention.

### Stability of ADP

**Fig 4:**
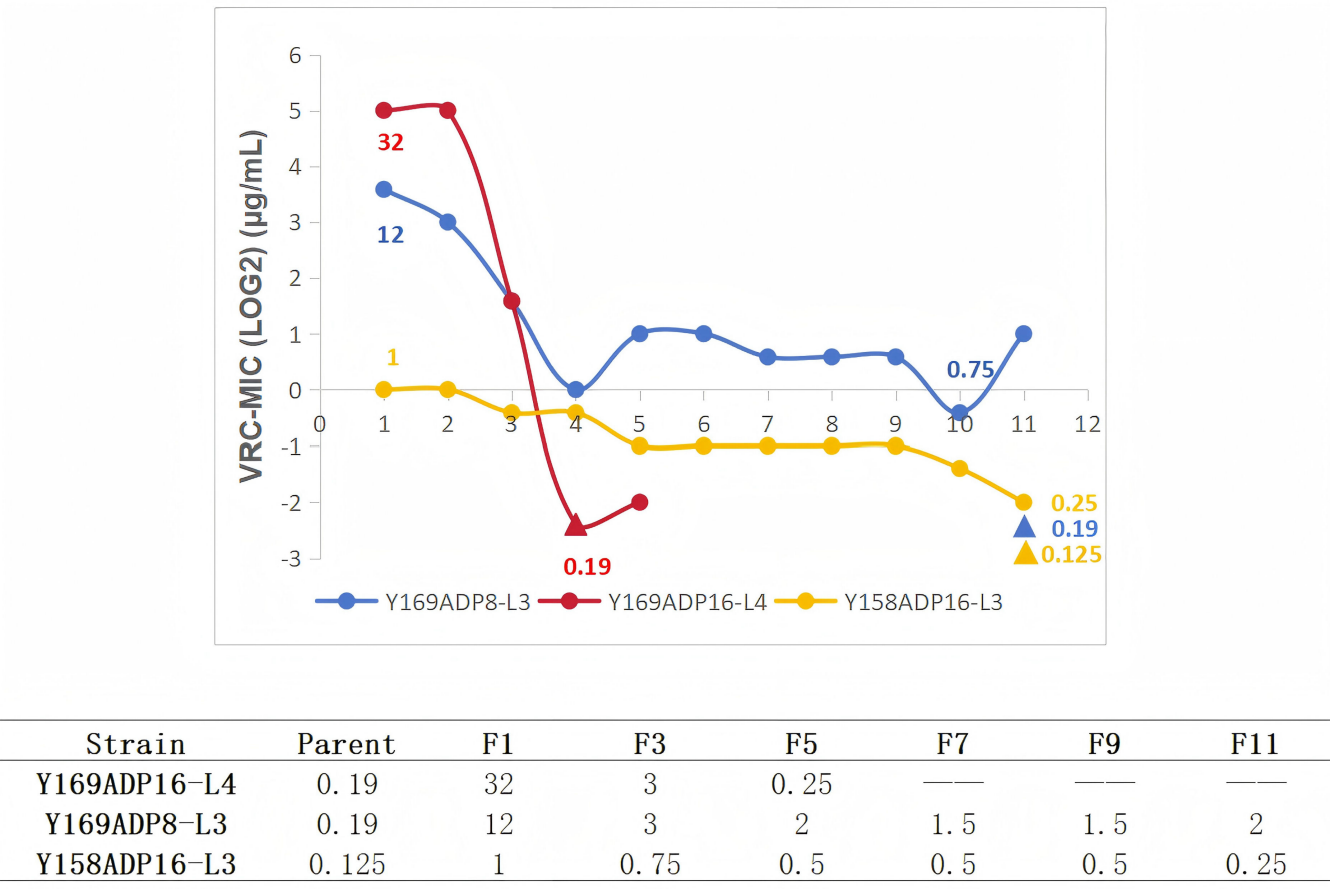
Changes in VRC MIC values of Y169ADP8-L3, Y158ADP16-L3, and Y169ADP16-L4 after serial passaging on drug-free SDA plates. *X*-axis represents the number of passages, and *Y*-axis represents the LOG2 value of VRC-MIC. The initial MIC values and the lowest detected MIC values of the strains are marked in μg/mL. ▲ represents the MIC value of the parent strain.

We evaluated the stability of VRC heteroresistance using Y169ADP8-L3, which is tolerant to 8 µg/mL, along with Y158ADP16-L3 and Y169ADP16-L4, both tolerant to 16 µg/mL. Results revealed that after serial passaging on drug-free SDA plates, the isolates gradually lost their acquired resistance to voriconazole, and the time required for resistance loss varied among different strains (Fig. 4). Specifically, Y169ADP16-L4 rapidly regained its initial sensitivity after only 4 passages (8 days) on drug-free SDA plates, whereas Y169ADP8-L3 and Y158ADP16-L3 remained more resistant than the original strain even after 11 passages (22 days). Due to the limited number of passages, the exact time required for these two strains to fully revert to their initial sensitivity was not determined.

## DISCUSSION

The escalating global burden of antifungal resistance has emerged as a critical public health crisis, jeopardizing decades of advancements in critical care, transplant medicine, and oncology. Within this context, heteroresistance, a cryptic yet clinically consequential phenomenon characterized by the proliferation of pre-existing resistant subpopulations under drug pressure despite eradication of susceptible counterparts, has been increasingly implicated as a potential contributor to therapeutic failure. Although increasingly documented in *Candida* and *Aspergillus* species (31–33), the prevalence and clinical implications of heteroresistance remain entirely uncharted in *T. asahii*, a life-threatening opportunistic pathogen associated with high mortality in immunocompromised hosts. Addressing this knowledge gap, our study analyzes a nationwide, genotype-diverse cohort of *T. asahii* strains from the CHIF-NET surveillance program (23). This work represents the first systematic investigation of voriconazole heteroresistance in this species.

Our study showed that all the *T. asahii* isolates tested had low voriconazole MICs (average 0.038 µg/mL), with 95.2% classified as wild type, which was consistent with a previous report that voriconazole showed the strongest *in vitro* activity against *T. asahii* (34, 35). Notably, our work, for the first time, revealed that ‌all *T. asahii* isolates exhibited heteroresistance to voriconazole‌, with heteroresistant subpopulations appearing at frequencies ranging from 0.002% to 0.830%. This discrepancy arises because heteroresistant subpopulations exist at low frequencies or exhibit resistance only under specific conditions, such as prolonged exposure to antifungal agents or within certain host environments (36). Traditional susceptibility testing methods‌ (e.g., CLSI broth microdilution) may fail to detect heteroresistant subpopulations. These tests typically measure the MIC for the bulk population, potentially overlooking small subpopulations that can survive higher drug concentrations (36). Our findings challenge the clinical interpretation of current susceptibility testing for guiding long-term voriconazole therapy and emphasize the clinical importance of developing more accurate and convenient heteroresistance detection methods. The PAP assay is currently the most established method for detecting heteroresistance. However, it is difficult to implement in clinical practice due to its time-consuming and labor-intensive characteristics. Recently, Gautier et al. (37) proposed the “short PAP assay” and “single-cell assays,” which are simplified and faster methods for measuring heteroresistance. Additionally, Zhai et al. (38) developed a machine learning-based predictive model utilizing genomic features to characterize heteroresistance in *Candida* species. These methods provide new insights for the clinical detection of heteroresistance, but further research is still required.

Our study reveals that heteroresistance to VRC is a universal and conserved trait in *T. asahii*, observed across all clinical isolates regardless of genotype or geographic origin. However, we found significant strain-to-strain variation in heteroresistance levels (LHV 0.25–4 µg/mL), with 27.4% (17/62) of strains displaying high-level heteroresistance (LHV ≥ 1 µg/mL). The clinical significance of varying LHV in *T. asahii* currently remains unclear. However, in *C. neoformans*, previous studies have established a clear correlation between higher heteroresistance levels and increased virulence in animal models (36). This compelling evidence from a related fungal pathogen strongly suggests the need to investigate whether similar virulence-heteroresistance associations exist in *T. asahii*, which would have important implications for clinical management.

Heteroresistance has emerged as a critical determinant of antibiotic treatment failure *in vivo*, challenging not only conventional susceptibility testing but also therapeutic strategies. Studies demonstrated that resistant subpopulations, often undetectable by standard diagnostics, can rapidly dominate under drug pressure, leading to persistent or relapsing infections. Band and Weiss (23) highlighted that while colistin was effective in rescuing mice infected with a susceptible strain, those infected with colistin-heteroresistant isolates failed colistin therapy and were unable to survive. Similarly, in *C. glabrata*, Ben-Ami et al. (18) revealed that fluconazole-heteroresistant subpopulations are associated with the persistence of viable *C. glabrata* cells in mouse kidney tissue during fluconazole treatment. Zhai et al. (38) reported that micafungin heteroresistance causes prophylaxis failure in allo-HCT recipients and facilitates breakthrough *C. parapsilosis* infections. These studies collectively emphasize that heteroresistance is not merely a laboratory artifact but a clinically significant phenomenon with profound implications for antimicrobial efficacy, which strengthens the hypothesis that similar mechanisms may operate in *T. asahii*. Additionally, treatment failure may also be related to interactions between molecules, the immune status of the patients, the timing of antifungal treatment, or other factors. While our data do not yet establish a direct link to clinical failure, we provide foundational evidence that heteroresistance, a previously unrecognized trait in *T. asahii*, may complicate antifungal management. To elucidate the clinical implications of heteroresistance, further *in vivo* studies should prioritize multifaceted approaches, such as establishing murine models of disseminated trichosporonosis, to evaluate VRC heteroresistance and its impact on therapeutic efficacy.

Heteroresistance is a stress response mechanism developed by microorganisms to adapt to increasing drug concentrations, enhancing their survival under antimicrobial pressure. In this study, the heteroresistant subpopulations demonstrated remarkable adaptability, rapidly developing resistance after short-term VRC exposure, with 71.0% of isolates able to tolerate VRC concentrations up to 8 µg/mL. However, this heteroresistance is unstable, and the isolates will gradually lose their resistance ability after serial passages on drug-free SDA plates. Previous studies have suggested that heteroresistance may occur through two potential mechanisms: the first posits that heteroresistance is caused by resistance mutations (e.g., single nucleotide polymorphisms, insertions, and deletions) that are genetically stable but come with a high fitness cost. The second mechanism involves the inherent instability of the resistance mutations themselves, such as the tandem amplification of unstable genes (5, 17). This gene amplification can enhance the resistance of subpopulations but incurs a high fitness cost, leading to a rapid reduction in gene copy number and restoration of drug sensitivity once the selective pressure of the drug is removed. The rate of resistance loss in different strains may be related to the unique genetic mutations possessed by the resistant subpopulations (31). The transient nature of heteroresistance underscores the need for repeated susceptibility testing during treatment. Resistance may emerge during therapy but revert after drug withdrawal, complicating the interpretation of MIC results.

Furthermore, we found that isolates exposed to VRC acquired cross-resistance to POS, FLU, and ITR. As the resistance to VRC increased, the resistance to the other three antifungal agents also intensified. The observed cross-resistance pattern among azoles (VRC, POS, FLU, and ITR) suggests shared resistance mechanisms. Moreover, some studies have shown that heteroresistant subpopulations can also increase resistance to exogenous substances and antimicrobial agents (20, 30), while exhibiting higher virulence (32). The phenomenon of cross-resistance warrants clinical attention, as strains that develop resistance to voriconazole may rapidly acquire resistance to two or even all of the other antifungal agents, ultimately leading to treatment failure. Research has described the relevant molecular mechanisms of cross-resistance. For example, *C. albicans* chr5 aneuploidy caused by caspofungin exposure can result in cross-resistance to caspofungin, micafungin, and anidulafungin (33). In *C. neoformans*, chr1 disomy may confer cross-resistance to both azoles and 5-FC (39). However, the mechanisms of heteroresistance and cross-resistance in *T. asahii* remain to be further investigated.

The limitations of this study are as follows: (i) the number of *T. asahii* isolates tested was limited, and the strain sources did not include remote regions of China such as Tibet and Xinjiang, leading to an incomplete representation of genotypes. (ii) The study lacked an analysis of the potential correlation between recorded LHV and patients’ antifungal treatment outcomes. (iii) There was no assessment of the relationship between LHV and strain virulence. (iv) We observed heteroresistant clones with varying colony sizes, but the underlying molecular mechanisms remain unclear. We plan to conduct whole-genome sequencing of the strains and resistant subpopulations to analyze the specific mechanisms of heteroresistance in the future.

Taken together, the current study provides the first comprehensive characterization of voriconazole heteroresistance in *T. asahii*, significantly advancing our understanding of antifungal resistance dynamics in this emerging pathogen. Our findings challenge the reliability of conventional MIC-based therapeutic guidance for *T. asahii* infections. Future research must prioritize clinical outcome correlations, refined diagnostic approaches for detecting resistant subpopulations, and optimized treatment strategies that account for this adaptive phenomenon.

## Data Availability

The GenBank accession numbers for the IGS1 regions range from PV631267 to PV631328.

## References

[1] Li H, Guo M, Wang C, Li Y, Fernandez AM, Ferraro TN, Yang R, Chen Y. 2020. Epidemiological study of Trichosporon asahii infections over the past 23 years. Epidemiol Infect 148:e169. doi:10.1017/S0950268820001624 32703332 PMC7439294

[2] Padovan ACB, Rocha WP da S, Toti AC de M, Freitas de Jesus DF, Chaves GM, Colombo AL. 2019. Exploring the resistance mechanisms in Trichosporon asahii: triazoles as the last defense for invasive trichosporonosis. Fungal Genet Biol 133:103267. doi:10.1016/j.fgb.2019.103267 31513917

[3] Sugita T, Nishikawa A, Ichikawa T, Ikeda R, Shinoda T. 2000. Isolation of Trichosporon asahii from environmental materials . Med Mycol 38:27–30. doi:10.1080/mmy.38.1.27.30 10746224

[4] Colombo AL, Padovan ACB, Chaves GM. 2011. Current knowledge of Trichosporon spp. and trichosporonosis. Clin Microbiol Rev 24:682–700. doi:10.1128/CMR.00003-11 21976604 PMC3194827

[5] Nicoloff H, Hjort K, Levin BR, Andersson DI. 2019. The high prevalence of antibiotic heteroresistance in pathogenic bacteria is mainly caused by gene amplification. Nat Microbiol 4:504–514. doi:10.1038/s41564-018-0342-0 30742072

[6] Gross JW, Kan VL. 2008. Trichosporon asahii infection in an advanced AIDS patient and literature review. Aids 22:793–795. doi:10.1097/QAD.0b013e3282f51ecc 18356615

[7] Bayramoglu G, Sonmez M, Tosun I, Aydin K, Aydin F. 2008. Breakthrough Trichosporon asahii fungemia in neutropenic patient with acute leukemia while receiving caspofungin. Infection 36:68–70. doi:10.1007/s15010-007-6278-6 17882360

[8] Liu X-Z, Wang Q-M, Theelen B, Groenewald M, Bai F-Y, Boekhout T. 2015. Phylogeny of tremellomycetous yeasts and related dimorphic and filamentous basidiomycetes reconstructed from multiple gene sequence analyses. Stud Mycol 81:1–26. doi:10.1016/j.simyco.2015.08.001 26955196 PMC4777771

[9] Arendrup MC, Boekhout T, Akova M, Meis JF, Cornely OA, Lortholary O. 2014. ESCMID and ECMM joint clinical guidelines for the diagnosis and management of rare invasive yeast infections. Clin Microbiol Infect 20:76–98. doi:10.1111/1469-0691.12360 24102785

[10] Chen SC-A, Perfect J, Colombo AL, Cornely OA, Groll AH, Seidel D, Albus K, de Almedia JN Jr, Garcia-Effron G, Gilroy N, et al.. 2021. Global guideline for the diagnosis and management of rare yeast infections: an initiative of the ECMM in cooperation with ISHAM and ASM. Lancet Infect Dis 21:e375–e386. doi:10.1016/S1473-3099(21)00203-6 34419208

[11] Alexander HE, Leidy G. 1947. Mode of action of streptomycin on type b H. influenzae: I. Origin of resistant organisms. J Exp Med 85:329–338. doi:10.1084/jem.85.4.329 19871618 PMC2135617

[12] El-Halfawy OM, Valvano MA. 2015. Antimicrobial heteroresistance: an emerging field in need of clarity. Clin Microbiol Rev 28:191–207. doi:10.1128/CMR.00058-14 25567227 PMC4284305

[13] Ferreira GF, Santos JRA, Costa MC da, Holanda RA de, Denadai ÂML, Freitas GJC de, Santos ÁRC, Tavares PB, Paixão TA, Santos DA. 2015. Heteroresistance to itraconazole alters the morphology and increases the virulence of Cryptococcus gattii. Antimicrob Agents Chemother 59:4600–4609. doi:10.1128/AAC.00466-15 26014951 PMC4505268

[14] Moreira I de MB, Cortez ACA, de Souza ÉS, Pinheiro SB, de Souza Oliveira JG, Sadahiro A, Cruz KS, Matsuura ABJ, Melhem M de SC, Frickmann H, de Souza JVB. 2022. Investigation of fluconazole heteroresistance in clinical and environmental isolates of Cryptococcus neoformans complex and Cryptococcus gattii complex in the state of Amazonas, Brazil . Med Mycol Open Access 60. doi:10.1093/mmy/myac005 35084497

[15] Ferreira GF, Santos DA. 2017. Heteroresistance and fungi. Mycoses 60:562–568. doi:10.1111/myc.12639 28660647

[16] Andersson DI, Nicoloff H, Hjort K. 2019. Mechanisms and clinical relevance of bacterial heteroresistance. Nat Rev Microbiol 17:479–496. doi:10.1038/s41579-019-0218-1 31235888

[17] Stone NRH, Rhodes J, Fisher MC, Mfinanga S, Kivuyo S, Rugemalila J, Segal ES, Needleman L, Molloy SF, Kwon-Chung J, Harrison TS, Hope W, Berman J, Bicanic T. 2019. Dynamic ploidy changes drive fluconazole resistance in human cryptococcal meningitis. J Clin Invest 129:999–1014. doi:10.1172/JCI124516 30688656 PMC6391087

[18] Ben-Ami R, Zimmerman O, Finn T, Amit S, Novikov A, Wertheimer N, Lurie-Weinberger M, Berman J. 2016. Heteroresistance to fluconazole is a continuously distributed phenotype among Candida glabrata clinical strains associated with in vivo persistence. MBio 7:e00655-16. doi:10.1128/mBio.00655-16 27486188 PMC4981708

[19] Chang YC, Khanal Lamichhane A, Kwon-Chung KJ. 2018. Cryptococcus neoformans, unlike Candida albicans, forms aneuploid clones directly from uninucleated cells under fluconazole stress. MBio 9:e01290-18. doi:10.1128/mBio.01290-18 30514783 PMC6282203

[20] Varma A, Kwon-Chung KJ. 2010. Heteroresistance of Cryptococcus gattii to fluconazole. Antimicrob Agents Chemother 54:2303–2311. doi:10.1128/AAC.00153-10 20385871 PMC2876399

[21] Yamazumi T, Pfaller MA, Messer SA, Houston AK, Boyken L, Hollis RJ, Furuta I, Jones RN. 2003. Characterization of heteroresistance to fluconazole among clinical isolates of Cryptococcus neoformans. J Clin Microbiol 41:267–272. doi:10.1128/JCM.41.1.267-272.2003 12517859 PMC149577

[22] van Hal SJ, Wehrhahn MC, Barbagiannakos T, Mercer J, Chen D, Paterson DL, Gosbell IB. 2011. Performance of various testing methodologies for detection of heteroresistant vancomycin-intermediate Staphylococcus aureus in bloodstream isolates. J Clin Microbiol 49:1489–1494. doi:10.1128/JCM.02302-10 21270232 PMC3122872

[23] Band VI, Weiss DS. 2019. Heteroresistance: a cause of unexplained antibiotic treatment failure? PLoS Pathog 15:e1007726. doi:10.1371/journal.ppat.1007726 31170271 PMC6553791

[24] Wang H, Xiao M, Chen SC-A, Kong F, Sun Z-Y, Liao K, Lu J, Shao H-F, Yan Y, Fan H, Hu Z-D, Chu Y-Z, Hu T-S, Ni Y-X, Zou G-L, Xu Y-C. 2012. In vitro susceptibilities of yeast species to fluconazole and voriconazole as determined by the 2010 National China Hospital Invasive Fungal Surveillance NET (CHIF-NET) study. J Clin Microbiol 50:3952–3959. doi:10.1128/JCM.01130-12 23035204 PMC3502960

[25] De Pauw B, Walsh TJ, Donnelly JP, Stevens DA, Edwards JE, Calandra T, Pappas PG, Maertens J, Lortholary O, Kauffman CA, et al.. 2008. Revised definitions of invasive fungal disease from the European Organization for Research and Treatment of Cancer/Invasive Fungal Infections Cooperative Group and the National Institute of Allergy and Infectious Diseases Mycoses Study Group (EORTC/MSG) Consensus Group. Clin Infect Dis 46:1813–1821. doi:10.1086/588660 18462102 PMC2671227

[26] Sugita T, Nakajima M, Ikeda R, Matsushima T, Shinoda T. 2002. Sequence analysis of the ribosomal DNA intergenic spacer 1 regions of Trichosporon species. J Clin Microbiol 40:1826–1830. doi:10.1128/JCM.40.5.1826-1830.2002 11980969 PMC130926

[27] Espinel-Ingroff A, Pfaller M, Erwin ME, Jones RN. 1996. Interlaboratory evaluation of Etest method for testing antifungal susceptibilities of pathogenic yeasts to five antifungal agents by using casitone agar and solidified RPMI 1640 medium with 2% glucose. J Clin Microbiol 34:848–852. doi:10.1128/jcm.34.4.848-852.1996 8815095 PMC228904

[28] Maxwell MJ, Messer SA, Hollis RJ, Boyken L, Tendolkar S, Diekema DJ, Pfaller MA. 2003. Evaluation of Etest method for determining fluconazole and voriconazole MICs for 279 clinical isolates of Candida species infrequently isolated from blood. J Clin Microbiol 41:1087–1090. doi:10.1128/JCM.41.3.1087-1090.2003 12624034 PMC150324

[29] Berman J, Krysan DJ. 2020. Drug resistance and tolerance in fungi. Nat Rev Microbiol 18:319–331. doi:10.1038/s41579-019-0322-2 32047294 PMC7231573

[30] Yang F, Gritsenko V, Lu H, Zhen C, Gao L, Berman J, Jiang Y. 2021. Adaptation to fluconazole via aneuploidy enables cross-adaptation to amphotericin B and flucytosine in Cryptococcus neoformans. Microbiol Spectr 9:e0072321. doi:10.1128/Spectrum.00723-21 34585947 PMC8557924

[31] Kuang Q, He D, Sun H, Hu H, Li F, Li W, Hu G, Wu H, Yuan L. 2020. R^93^P substitution in the PmrB HAMP domain contributes to colistin heteroresistance in Escherichia coli isolates from swine . Antimicrob Agents Chemother 64:11. doi:10.1128/AAC.01509-20 PMC7577138 32868331

[32] Sionov E, Chang YC, Garraffo HM, Kwon-Chung KJ. 2009. Heteroresistance to fluconazole in Cryptococcus neoformans is intrinsic and associated with virulence. Antimicrob Agents Chemother 53:2804–2815. doi:10.1128/AAC.00295-09 19414582 PMC2704677

[33] Husain F, Yadav A, Sah SK, Hayes JJ, Rustchenko E. 2022. Candida albicans strains adapted to caspofungin due to aneuploidy become highly tolerant under continued drug pressure. Microorganisms 11:23. doi:10.3390/microorganisms11010023 36677315 PMC9866909

[34] Guo L-N, Yu S-Y, Hsueh P-R, Al-Hatmi AMS, Meis JF, Hagen F, Xiao M, Wang H, Barresi C, Zhou M-L, de Hoog GS, Xu Y-C. 2019. Invasive infections due to Trichosporon: species distribution, genotyping, and antifungal susceptibilities from a multicenter study in China. J Clin Microbiol 57:e01505-18. doi:10.1128/JCM.01505-18 30463892 PMC6355529

[35] Francisco EC, de Almeida Junior JN, de Queiroz Telles F, Aquino VR, Mendes AVA, de Andrade Barberino MGM, de Tarso O. Castro P, Guimarães T, Hahn RC, Padovan ACB, Chaves GM, Colombo AL. 2019. Species distribution and antifungal susceptibility of 358 Trichosporon clinical isolates collected in 24 medical centres. Clin Microbiol Infect 25:909. doi:10.1016/j.cmi.2019.03.026 30991116

[36] Su Y, Li Y, Yi Q, Xu Y, Sun T, Li Y. 2025. Insight into the mechanisms and clinical relevance of antifungal heteroresistance. J Fungi 11:143. doi:10.3390/jof11020143 PMC11856953 39997437

[37] Gautier C, Maciel EI, Ene IV. 2024. Approaches for identifying and measuring heteroresistance in azole-susceptible Candida isolates. Microbiol Spectr 12:e0404123. doi:10.1128/spectrum.04041-23 38483474 PMC10986555

[38] Zhai B, Liao C, Jaggavarapu S, Tang Y, Rolling T, Ning Y, Sun T, Bergin SA, Gjonbalaj M, Miranda E, Babady NE, Bader O, Taur Y, Butler G, Zhang L, Xavier JB, Weiss DS, Hohl TM. 2024. Antifungal heteroresistance causes prophylaxis failure and facilitates breakthrough Candida parapsilosis infections. Nat Med 30:3163–3172. doi:10.1038/s41591-024-03183-4 39095599 PMC11840754

[39] Zhang Z, Sun L, Fu B, Deng J, Jia C, Miao M, Yang F, Cao Y, Yan T. 2024. Aneuploidy underlies brefeldin A-induced antifungal drug resistance in Cryptococcus neoformans. Front Cell Infect Microbiol 14:1397724. doi:10.3389/fcimb.2024.1397724 38966251 PMC11222406

